# Classification of cognitive ability of healthy older individuals using resting-state functional connectivity magnetic resonance imaging and an extreme learning machine

**DOI:** 10.1186/s12880-024-01250-3

**Published:** 2024-03-26

**Authors:** Shiying Zhang, Manling Ge, Hao Cheng, Shenghua Chen, Yihui Li, Kaiwei Wang

**Affiliations:** 1 State Key Laboratory of Reliability and Intelligence of Electrical Equipment, Hebei University of Technology, Tianjin, China; 2 Hebei Province Key Laboratory of Electromagnetic Field and Electrical Apparatus Reliability, Hebei University of Technology, Tianjin, China; 3 School of Health Sciences and Biomedical Engineering, Hebei University of Technology, Tianjin, China; 4 Tianjin Hebei University of Technology, 5340 Xiping Road, Beichen District, Tianjin, 300130 China; 5 Hebei University of Technology, 8 Guangrong Road, Hongqiao District, Tianjin, 300130 China

**Keywords:** Resting-state FC, Older, Cognitive test score, Extreme learning machine, 10-fold cross-validation

## Abstract

**Background:**

Quantitative determination of the correlation between cognitive ability and functional biomarkers in the older brain is essential. To identify biomarkers associated with cognitive performance in the older, this study combined an index model specific for resting-state functional connectivity (FC) with a supervised machine learning method.

**Methods:**

Performance scores on conventional cognitive test scores and resting-state functional MRI data were obtained for 98 healthy older individuals and 90 healthy youth from two public databases. Based on the test scores, the older cohort was categorized into two groups: excellent and poor. A resting-state FC scores model (rs-FCSM) was constructed for each older individual to determine the relative differences in FC among brain regions compared with that in the youth cohort. Brain areas sensitive to test scores could then be identified using this model. To suggest the effectiveness of constructed model, the scores of these brain areas were used as feature matrix inputs for training an extreme learning machine. classification accuracy (CA) was then tested in separate groups and validated by N-fold cross-validation.

**Results:**

This learning study could effectively classify the cognitive status of healthy older individuals according to the model scores of frontal lobe, temporal lobe, and parietal lobe with a mean accuracy of 86.67%, which is higher than that achieved using conventional correlation analysis.

**Conclusion:**

This classification study of the rs-FCSM may facilitate early detection of age-related cognitive decline as well as help reveal the underlying pathological mechanisms.

## Introduction

Currently in many countries, as the proportion of older population increases, health problems of the older are getting more and more attention, among which, cognitive decline is a key factor that threatens the quality of life of the older. An age-related decline in cognitive abilities may be an early indicator of neurodegenerative and psychiatric disorders such as Alzheimer’s disease (AD) [1]. Cognitive evaluation of the older at an early stage and timely interventions will help reduce the rate of deterioration. Traditionally, older adults cognitive abilities have been assessed by traditional cognitive scale tests designed, licensed, and measured by psychologists [2, 3]. However, the relationship between a healthy aging brain and cognitive performance is unclear, and the assessment of cognitive ability is still traditional to some extent. Comprehensive testing is time-consuming, complex and subjective, which affects the accurate evaluation of cognitive functions of the older. At present, functional magnetic resonance imaging (fMRI) could provide objective measurements of cognitive functions by the FC computing [4–6]. Furthermore, fMRI evaluations can be conducted both during tasks engaging specific neural networks or in the resting state to provide an unbiased assessment of network activity.

Resting-state fMRI (rs-fMRI) can reveal the FC of the whole brain by measuring the spatiotemporal correlations in regional brain activity established [7–9]. In addition, rs-fMRI is noninvasive and objective, and in contrast to neuropsychiatric tests and task-dependent fMRI, can be conducted quickly (in less than 15 min), thereby permitting larger-scale screening of older populations free of outcome variations conferred by different cognitive tasks and subject scoring criteria. The conventional FC has been defined by Pearson correlation and widely used. A growing number of studies have used fMRI techniques to construct FC matrices as a tool for early cognitive diagnostic sensitization of cognition in older adults, for example, Cera et al. calculated FC matrices with anterior cingulate gyrus brain regions as seed points in healthy older adults and patients with mild cognitive impairment (MCI), and found that FC matrices levels were significantly increased in the ventral part of the anterior cingulate cortex with bilateral caudate and ventral medial prefrontal cortex in healthy older adults as compared to those with MCI [10].

Now, several well-known fMRI-based consortium projects have supplied public fMRI datasets to estimate the FC matrices, including the International Consortium for Brain Mapping established in 1992; the United States Human Connectome Project (HCP), which provided a stable brain function computing template. And the Brain Genomics Superstructure Project (GSP) established by Harvard Medical School in 2014 [11]. As a pioneer, a profound public cohort, Leading Eigenvector Dynamics Analysis (LEiDA) [12], provided the rs-fMRI data and the psychometric scale tests performances in healthy aging. In particular, the emerging of multi-site homogenization in recent years, such as the ComBat multi-site homogenization algorithm, can get rid off the heterogeneous functional connection values across different datasets, therefore, it is popular to make use of these plentiful data sources in the world [13].

**Fig. 1:**
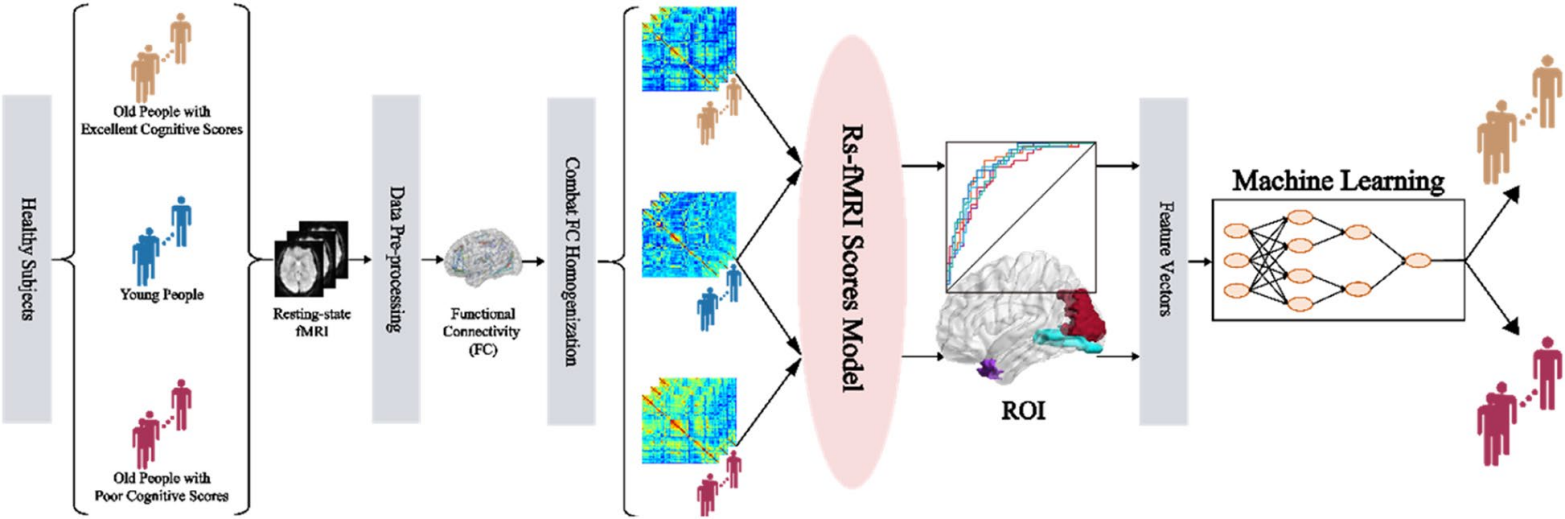
Study flowchart

Previous studies have found that the youth exhibit an optimal network framework for language processing characterized by highly integrated local networks with strong FC matrices and weaker FC matrices between networks [14], whereas older individuals with reduced language comprehension demonstrate a suboptimal network structure characterized by stronger FC matrices between networks [15]. These findings may help identify older individuals at risk of progressive cognitive impairment, thereby facilitating timely intervention. At present, a combination of structural MRI and machine learning is used to classify older individuals with cognitive impairment [16, 17]. However, there are few models to identify quantitative regional FC biomarkers sensitive to cognitive scores among healthy older individuals. Here, we propose the rs-FCSM to estimate the degree of FC deviation between an older individual and a youth cohort from the connectome with the help of homogenization technology, for purpose of improving conventional FC computing of Pearson correlation up to a high level and taking advantage of two-site valuable connectome datasets. To depict the ability of the rs-FCSM, its combination with a machine learning model was employed to classify older individuals with excellent or poor cognition. The study flowchart is shown in Fig. 1.

## Material and methods

### Experimental dataset

#### Healthy older groups

Older individuals were selected from a public dataset LeiDA derived from a cohort study involving 1,051 healthy Portuguese older individuals aged > 50 years, who were subjected to nine neuropsychological tests [12]. The tests were approved by certified psychologists. Furthermore, Santos et al. used Principal Component Analysis applied to all neuropsychological data, two main dimensions of cognitive performance were identified, one being related to memory and the other to general executive functioning. Cluster analysis based on the scores of these two dimensions and those of other related variables such as health status revealed four functional categories: C1 (excellent cognition) > C2 > C3 > C4 (poor cognition) [18]. Ninety-eight subjects were randomly selected from the C1 and C4 groups to undergo rs-fMRI scans: 55 subjects with excellent cognitive scores (excellent group) and 43 with poor cognitive scores (poor group). The excellent cognition level (C1) and poor cognition level (C4) among the four cognitive levels of healthy older adults were chosen as the subjects of the study because it was considered that the brain regions with functional differences were better highlighted in the two larger cognitive level differences.

Prior to the acquisition, participants were instructed to remain still with eyes closed, not to fall asleep and not to think about anything. The fMRI acquisition was performed using a clinical approved 1.5T Siemens Magnetom Avanto (Siemens Medical Solutions, Erlangen, Germany) MRI scanner with a 12-channel receive-only head coil at Hospital de Braga (Portugal). A blood oxygenation level-dependentsensitive (BLOD) echo-planar imaging (EPI) sequence was used with the following parameterization: 30 axial slices, time of repetition (TR)/ time of echo (TE) = 2000/30 ms, flip angle (FA) = 90°, voxel size = 3.5 mm^3^, field of view (FOV) = 134.4 mm^2^.

This study was performed in accordance with the Declaration of Helsinki (59^th^ amendment), and all subjects provided written informed consent [18].

#### Healthy young group

Ninety healthy youth aged 18–35 years were selected from > 1500 subjects in the GSP database [11]. Since the subject of the study is Portuguese and the reference subject is American, this could introduce potential differences in the calculations of the model. On the one hand, the researcher considered that in the calculations, all the Portuguese did the calculations with every American, while in the end it was the Portuguese of both cognitive levels who did the analysis of variance between them, so there was the possibility that the effects of the different ethnicities were canceled out in the process; on the other hand, in order to reduce the heterogeneity, the Combat algorithm was used to de-heterogenize the two categories. As a result, young Americans were finally selected as the reference for calculating the scores.

The dataset for each participant in the GSP database includes 1) basic demographic and health information before and after MRI scans, 2) structural and functional MRI scans. Before scanning, all neurological functions, psychological evaluations, and language comprehension scores are higher than the population median. Subjects with mental illness or a history of mental illness were excluded.

All imaging data were collected on matched 1.5T Siemens Magnetom Avanto (Siemens Healthcare, Erlangen, Germany) at Harvard University and Massachusetts General Hospital using the vendor-supplied 12-channel phased-array head coil. Functional imaging data were acquired using a gradient-echo EPI sequence sensitive to BOLD contrast. EPI parameters were as follows: TR = 3,000 ms, TE = 30 ms, FA = 85°, voxel size = 3 mm^3^, FOV = 216 mm^2^. During BOLD data collection, participants were instructed to remain still, stay awake.

This study was performed in accordance with the Declaration of Helsinki (59^th^ amendment), and all subjects provided written informed consent [11].

### Data pre-processing

#### Healthy older groups

All of the rs-fMRI data preprocessing of the older has been completed by the data disclosure party before open. These rs-fMRI data were pre-processed in the following steps using the FMRIB software library (FSL V5.07) [19]. (1) The first five acquisition time series were removed to stabilize the signal. (2) Slice timing correction was performed. (3) MCFLIRT software was used to perform rigid body alignment motion correction [20]. (4) Skull stripping with the Brain Extraction Tool. (5) Nonlinear normalization through continuous rigid body registration was performed. FLIRT software was employed to obtain the structure from the original space, and nonlinear registration was performed in Montreal Neurological Institute standard space, followed by re-sampling to an isotropic voxel size of 2 mm^3^. (6) Linear regression was conducted to remove motion parameters, mean cerebrospinal fluid signals, and white matter (WM) signals. (7) Finally, band-pass time filtering of regression residuals (0.01‒0.08 Hz) was conducted [18]. The database only discloses data files of the older after matching the automatic anatomical labeling (AAL, Version No.1) atlas [21].

#### Healthy young group

The rs-fMRI data in the young group were preprocessed by using the software FMRIB [19]. The processing procedure and quality inspection (framewise displacement [FD] < 0.2 mm) were conducted at Martinos Center for Medical Imaging, Harvard Medical School, USA. The processing station was Intel Xeon Sliver 4112 × 16 with the operating system of Centos 7.6, and the preprocessing time was about 15 h for each subject.

Data preprocessing was performed as follows: (1) Removal of the first four acquisition time series to stabilize the signal; (2) Slice timing correction using SPM 12 (http://www.fil.ion.ucl.ac.uk/spm/); (3) Rigid body correction for head motion using the FSL package; (4) Normalization for global mean signal intensity across runs and registration of the signal to the standard space of Montreal Neurological Institute; (5) Band-pass temporal filtering (0.01 Hz-0.08 Hz) to reduce high-frequency signal interference; (6) Matching of the rfMRI data of each subject with the AAL Atlas.

**Fig. 2:**
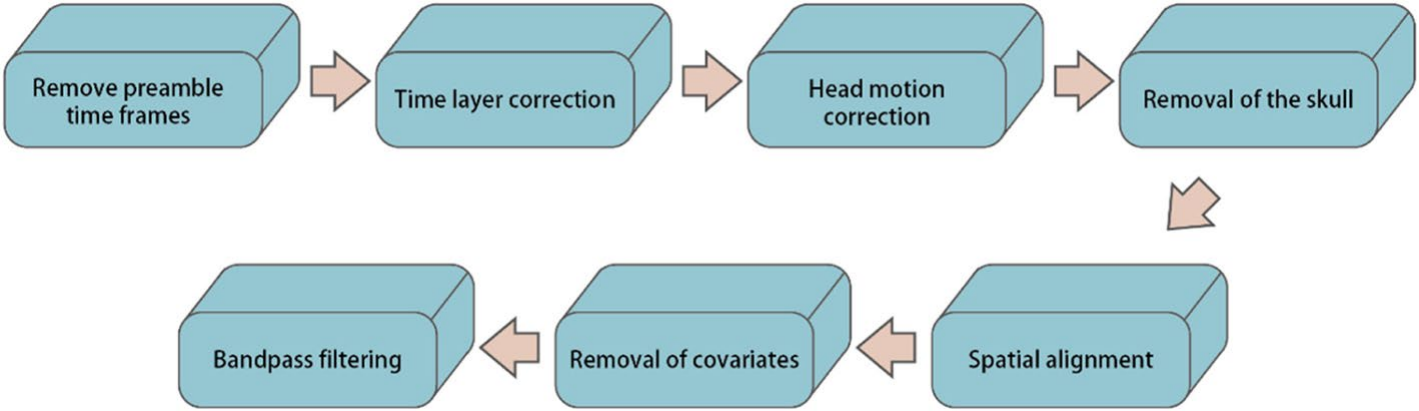
Flowchart of pre-processing

The flowchart of the preprocessing is shown in Fig. 2.

### Post-processing

#### Functional connectivity (FC)

Conventionally, the coefficient of Pearson correlation is usually used as a FC to study the degree of dependence between time series on two brain areas during resting state, with an assumption of static BOLD signal, that all-time series of BOLD signals would participate in a FC estimation. The coefficient is between—1 and + 1. Being greater than zero means positively correlated (i.e., if the activity of one brain area excited, the activity of another brain area would be excited, and vice versa). In reversal, being less than zero means negatively correlated, that is, if the activity of one brain area excited, the activity of another brain area would be inhibited; Being 1 means self- correlation, or being zero means no correlation between two brain areas. The absolute value of the coefficient represents for the degree of correlation. The greater the value, the stronger the correction is, and vice versa.

Here, the functional connection of Pearson was estimated between two brain regions to form the correlation coefficient matrix. Since each subject’s brain was divided into 116 brain regions by the AAL atlas, a 116 × 116 matrix were formed. Cohort characteristics are shown by subject-averaged FC matrices for two older groups, such as excellent group and poor group.

#### Combat multi-center data homogenization

To address the issue of central heterogeneity among the data from different databases, the technology of ComBat multi-center data homogenization will be performed among the databases FC matrices, for purpose of getting rid off the data heterogeneity between multiple connectome centrals to homogenize them. The triangular connection values on the FC matrices are arranged in a matrix transverse order, and each matrix is integrated into a 1*6670 connection vector. The ComBat multi-site homogenization algorithm takes all connection vectors as inputs and performs homogenization calculation [22]. Its core calculation is shown in formula (1):

1 $$\begin{array}{c}{y}_{i,j,v}^{ComBat}=\frac{{y}_{i,j,v}-\widehat{{a}_{v}}-{X}_{i,j}*\widehat{{\beta }_{v}}-{\gamma }_{i,v}^{*}}{{\delta }_{i,v}^{*}}+\widehat{{a}_{v}}+{\varvec{X}}_{\varvec{i},\varvec{j}}*\widehat{{\beta }_{v}}\end{array}$$

Where, *i* is the center number, *j* is the subject number, *v* is the vector number within a single subject, *y*_*i,j,v*_ is the *v*-th value of vector of the subject *j* at center *i* without homogenization, and av represents the average of the *v*-th value of vector in all subjects, ***X***_***i,j***_ is the covariate design matrix, *β*_*v*_ is the regression coefficient vector corresponding to ***X*,***γ*_*i,v*_ and *δ*_*i,v*_ correspond to the additive and multiplicative effects of site connectivity, respectively. The parameter with “ʌ” represents the Bayesian estimation of the parameter, and the superscript with “*” on the parameter represents the empirical Bayesian estimation of the parameter.

After each connection vector was calculated by the ComBat homogenization formula, the new connection vector could be refreshed to the FC matrix according to the original matrix transverse order to complete the multi-center homogenization and the FC strength was derived for a single mean value at each brain region. A toolbox for surfaces, nodes, and edges in BrainNet Viewer software was employed to display FC networks [23].

#### Resting-state FC scores model (rs-FCSM)

To extract the functional neuroimaging markers sensitive to the scores of cognitive scale tests of the healthy aging, the rs-FCSM was constructed from the differences in regional FC values between each older individual and the youth cohort. In the model, the regional FC difference between area “*i*” in older brain relative to areas “*i*” in the youth brains is expressed as a connectome distinctiveness index (CDI).

2 $$CDI_{s,i}=\frac1{N-1}\sum\limits_{p=1}^N\left(1-corr\left(f_{s,i},f_{p,i}\right)\right)$$

Where *f*_*s,i*_ is the FC vector between the *i*-th brain region and other brain regions of the whole brain in the *s*-th older brain, *f*_*p,i*_ is the FC vector between the *i*-th brain region and other brain regions of the whole brain in the *p*-th youth brain, N is the number of healthy youth, and *CDI*_*s,i*_ is the value of the rs-FCSM of the *i*-th area in the *s*-th healthy older brain.

The *CDI*_*s,i*_ values of 98 healthy older and 90 healthy young adults were calculated by row according to formula (2), and finally collated to form a *CDI* of 98 × 116 for older brain (1 × 116 matrix for each individual) and a *CDI* of 90 × 116 for the young brain (1 × 116 matrix for each individual).

The distribution of *CDI* in the youth brain was evaluated using the equations:

3 $$mean\_CDI_i=\frac1N\sum\limits_{p=1}^NCDI_{p,i}$$

4 $$std\_CDI_i=sqrt\left(\frac1{N-1}\sum\limits_{p=1}^N\left(CDI_{p,i}-mean\_CDI_i\right)^2\right)$$

where *mean_CDI*_*i*_ represents the average *CDI*_*p,i*_ for N healthy youth (*N* = 90) and *std_CDI*_*i*_ is the standard deviation of *CDI*_*p,i*_ at the *i*-th brain area.

Relative to the healthy cohort, the *Z* score of *CDI*_*s,i*_ in older individual is defined as

5 $$Z_{s,i}=\frac1{std\_CDI_i}\left(CDI_{s,i}-mean\_CDI_i\right)$$

Where *Z*_*s,i*_ denotes the final value of the rs-FCSM of the *i*-th brain area of the *s*-th subject in older cohort.

This rs-FCSM can objectively estimate the FC deviation from the healthy youth for each AAL-labelled brain region, with higher values of the rs-FCSM indicating a greater degree of deviation in an older individual compared with young cohort. As 98 healthy older individuals were examined, 98 × 116 index matrices were formed.

#### Extraction of functional biomarker regions by the rs-FCSM

First, the difference between the mean values of the values of the rs-FCSM of each brain region of the two older groups was calculated, and recorded as Difference_mean. Second, the sum of the standard deviations of the values of the rs-FCSM of each brain region of the two older groups was calculated, and recorded as Sum_std. Finally, the determination of theregion of interest (ROl) needs to meet two conditions at the same time, (1) ROIs belong to the top 10% of the brain regions (12 ROIs) with the largest Difference_mean; (2) ROIs do not belong to the top 10% of the brain regions with the largest Sum_std (12 ROIs).The extracted ROIs were regarded as the functional biomarkers regions sensitive to cognitive scores of the older and were projected to the cerebral cortex for visualization by the BrainNet viewer software [23].

To validate ROIs extracted by the above method, two kinds of analysis, such as a sensitivity analysis and a strict inter-group statistic, were employed. The sensitivity analysis could display ROI character by the receiver operating characteristic (ROC) curve with the help of SPSS software (IBM SPSS Statistics 21; USA). The values of the rs-FCSM in ROIs between the excellent group and the poor group were input into the sensitivity analysis. The farther above the reference line, the more sensitive ROIs to cognitive scores of the older. On the other hand, the significant difference of the values of the rs-FCSM in ROIs between the excellent group and the poor group were respectively shown by the *p* values (*p* ≤ 5%) with a strict statistics, e.g., two-sample *t*-test corrected by strict Bonferroni multiple comparisons (Mathworks Matlab 2018a; USA).

### Extreme learning machine

#### Feature vectors

The values of the rs-FCSM ROIs identified as functional biomarkers were then considered as feature vectors for the ELM input layer, with those from the poor group labeled as 1 and those from excellent the group labeled as 2.

#### ELM model

**Fig. 3:**
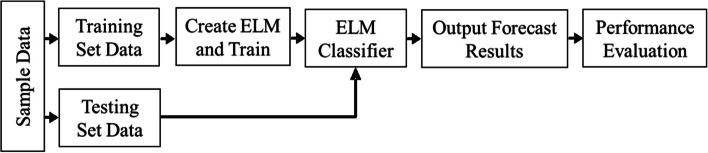
Flowchart of extreme learning machine (ELM) classifier

Compared with conventional artificial neural network models, an advantage of ELMs is that the model can randomly generate both the connection weights between the input layer and the hidden layer and the threshold values of neurons in the hidden layer. For training, ELM requires only a known number of neurons in the hidden layer for it to converge on a unique optimal solution. A flowchart of ELM construction, training, and testing is shown in Fig. 3.

The main steps of the ELM model are as follows, (1) training and testing datasets of sufficient size for ELM generalization and prediction performance were established, (2) the ELM was constructed and trained using the “elmtrain” function. A suitable number of hidden neurons was set for best performance, (3) simulation testing was conducted using the “elmredict” function to form the test set, (4) the accuracy of classification was then evaluated.

In ELM, the type parameter was set as 1 (Set to 1 to solve the classification problem and set to 0 to solve the regression problem), the number of neurons in the hidden layer was set to 500, and the activation function TF was set to “sig” type. Then, the *Z*_*s,i*_ were trained and simulated using ELM (Mathworks Matlab 2018a; USA).

#### N-fold cross-validation (*N* = 10)

In ELM, a N-fold cross-validation (*N* = 10) procedure was employed to test the accuracy of the algorithm as follows. The dataset was divided into 10 parts by setting 9 parts as the training data and 1 part as the testing data. Thus, classification accuracy could be assessed for each procedure. The mean accuracy over 10 iterations was utilized to estimate the accuracy of the algorithm.

## Results

### Conventional FC

**Fig. 4:**
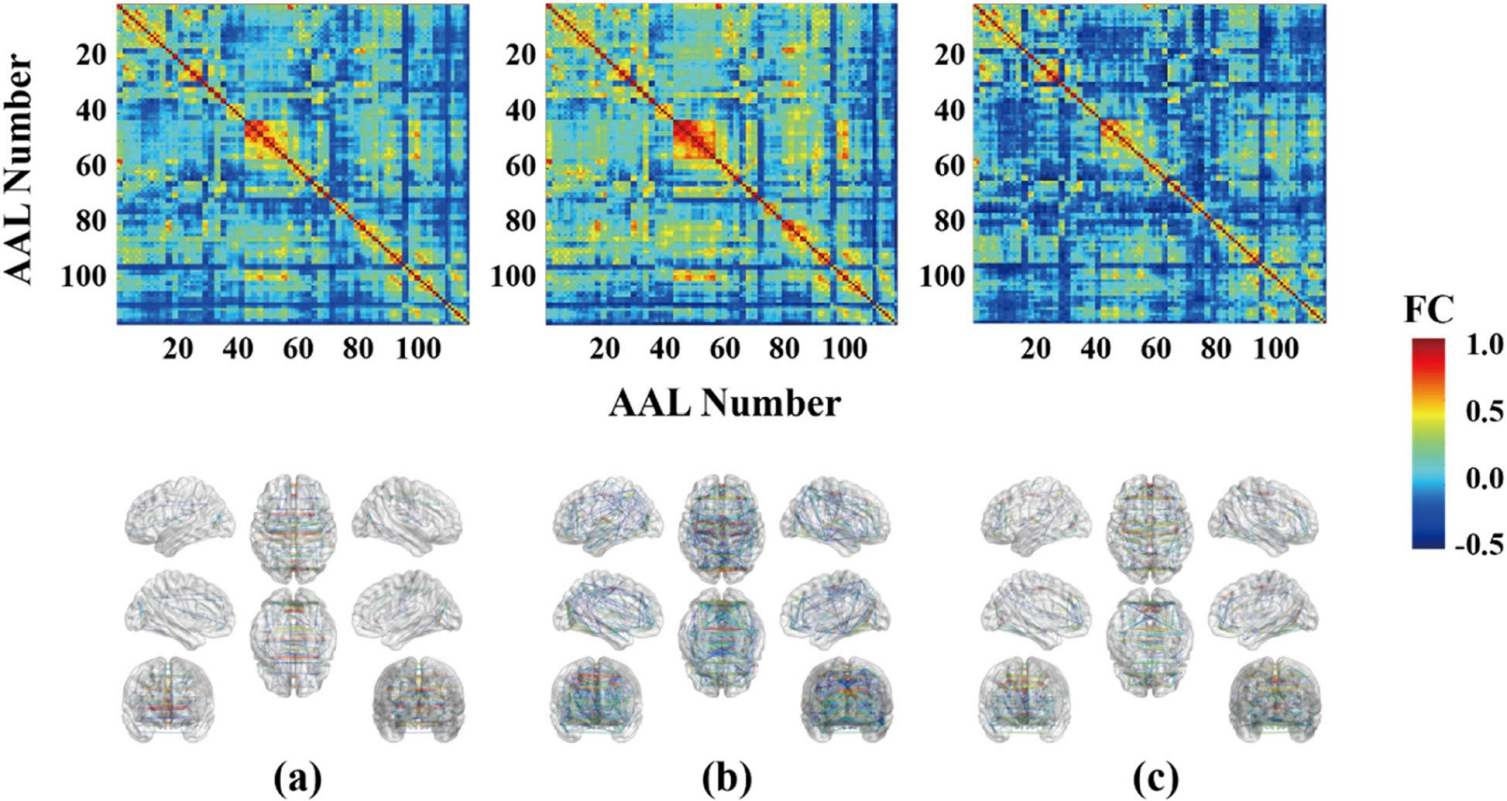
Difference in whole-brain functional connectivity (FC) between older individuals with excellent and poor cognitive test performance. **a** Average functional connectivity values for the 55 old people with excellent cognitive scores. **b** Average functional connectivity values for the 43 old people with poor cognitive scores. **c** Average functional connectivity values for 90 healthy youth. The functional connectivity values are displayed as color-coded matrices in the upper panels and as BrainNet Viewer networks in the below panels. The middle red line in the matrix is the self-correlation for each region

The subject-averaged FC matrices diagrams constructed from the rs-fMRI data of older individuals with excellent cognitive test scores resembled those constructed from the youth brains (Fig. 4). In contrast, the FC diagrams of older group with poor cognitive test scores exhibited substantial connectivity differences compared with the excellent group.

### Functional biomarkers estimated using the rs-FCSM

**Fig. 5:**
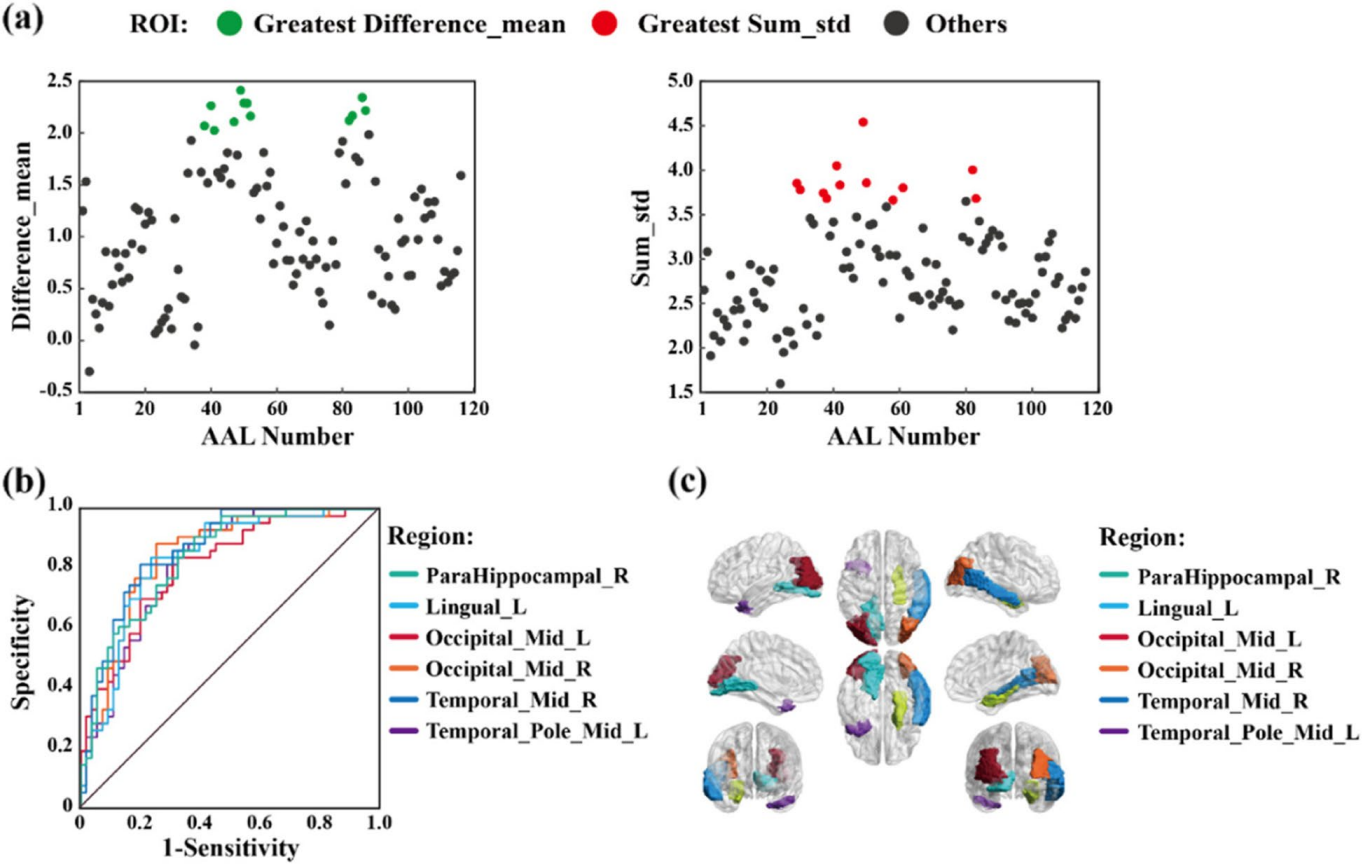
Six functional biomarkers (ROIs) of cognitive ability in healthy older. **a** Difference_mean and Sum_std of values of rs-FCSM for the whole brain. Regions with top 10% of greatest Difference_mean and Sum_std are respectively shown by green dots and red dots, the others are shown by black dots; **b** ROC curves of ROIs; **c** ROIs displayed by BrainNet Viewer

According to the previously defined rules for the determination of ROIs that are sensitive to cognitive scores (please “Extraction of functional biomarker regions by the rs-FCSM” section), the 12 brain regions (top 10% on AAL atlas) with the largest Difference_mean and Sum_std were counted in Fig. 5a, therefore, six sensitive ROIs were extracted to cognitive scores. These six brain regions were validated by their ROC curves above the reference lines in Fig. 5b, and the significance values with two sample *t*-test under Bonferroni multiple comparisons in Table 1 (*p* <  < 5%). In Fig. 5c, six brain regions, e.g., AAL40 (ParaHippocampal_R), AAL47 (Lingual_L), AAL51 (Occipital_Mid_R), AAL52 (Occipital_Mid_L), AAL86 (Temporal_Mid_R) and AAL87 (Temporal_Pole_Mid_L), were highlighted by BrainNet Viewer.

**Table 1:** Validation for ROIs by two sample t-test under Bonferroni multiple comparisons

AAL area	Significance (*p*-value)
ParaHippocampal_R (AAL40)	5.9193*10^–9^
Lingual_L (AAL47)	8.3207*10^–8^
Occipital_Mid_L (AAL51)	7.2708*10^–7^
Occipital_Mid_R (AAL52)	9.3708*10^–8^
Temporal_Mid_R (AAL86)	5.0471*10^–7^
Temporal_Pole_Mid_L (AAL87)	1.8694*10^–6^

It was resulted that the rs-FCSM could extract functional biomarkers of cognitive ability in healthy older adults, which were in parahippocampus, occipital lobe and temporal lobe regions.

### Classification using the ELM model

Each of ten parts is used in turn as the test set and the rest as the training set for ELM machine learning classification. Positive is classified as high cognitive level (C1) and negative is classified as low cognitive level (C4). The average accuracy after N-fold cross-validation is 86.67% in Table 2.

**Table 2:** Classification accuracy (CA) tested by 10-fold cross-validation

N	Subjects in test set	Sensitivity (%)	Specificity (%)	Accuracy (%)
1	10	80	80	80
2	10	83.33	100	90
3	10	80	100	90
4	10	100	75	90
5	10	66.67	75	70
6	10	100	80	90
7	10	100	100	100
8	10	100	75	90
9	9	80	75	77.78
10	9	80	100	88.89
Mean ± std		87 ± 12.01	86 ± 12.20	86.67 ± 8.43

## Discussion

### Sensitive brain areas identified by the rs-FCSM

FC analysis is widely applied to localize dysfunctional brain areas at a group level. For example, Zhang et al. reported that there were intergroup FC abnormalities in brain regions such as the hippocampus and middle temporal gyrus affected by mild cognitive impairment [24]. Moreover, Tang et al. developed a FC model and found that patients with AD had decreased FC in the caudate gyrus, limbic lobe, medial frontal gyrus (MFG), middle temporal gyrus, superior frontal gyrus, parietal/precuneus, inferior temporal gyrus, and posterior cingulate gyrus compared to healthy controls [25].

Our work extends such studies by demonstrating that FC differences associated with cognitive function are detectable among healthy older at an individual level. The functional biomarkers identified by the rs-FCSM also overlap with areas demonstrating structural abnormalities. For instance, using diffusion tensor imaging, Bolzenius et al. found cognitive impairment was related to reduce structural integrity of the temporal lobes [26]. Mokrisova et al. also found impaired path integration associated with reduced hippocampal volume and thinning of the entorhinal and parietal cortices, suggesting that the neurodegeneration of the medial temporal lobe and parietal cortex are quantitative indicators of disease status [27]. In contrast to studies with group level analysis, the rs-FCSM could distinguish excellent from poor cognition in healthy older individuals (Fig. 4 and Table 2).

### Greater sensitivity of the rs-FCSM compared with conventional FC models

The rs-FCSM developed in this study identified FC changes in single brain areas able to distinguish excellent from poor cognition among healthy older individuals with 86.67% accuracy, whereas conventional FC analysis (Fig. 4) using Pearson coefficients could distinguish cognitive performance only at the group level. Furthermore, doing same work by conventional FC model of Pearson correlation as this study, instead of the rs-FCSM, the highest classification accuracy was only 72.2%, the average classification accuracy was only 68.4% with a variance of 12.1%. The rs-FCSM performed better than conventional FC model in classify scores of cognitive scale tests in the healthy aging. Similarly, Benesty et al. reported 71.43% accuracy using Pearson correlation coefficients as FC metrics [28], and Goryawala et al. [29] found that neuropsychological scores and cortical volumes (temporal, parietal lobe, and cingulate gyrus) could distinguish early mild cognitive impairment (EMCI) from late mild cognitive impairment (LMCI) with 73.6% accuracy. Recently, Zhu et al. entered the FC of the hippocampus as features into a support vector machine and obtained a classification accuracy of 81.33% for healthy controls and older patients with mild cognitive impairment [30]. Table 3 compares several cognitive classification methods, and it can be seen that the model proposed in this paper has a good classification value. Thus, the rs-FCSM achieves better classification accuracy among healthy older individuals with excellent or poor cognitive ability than current classification models, which might be potential to replace the traditional scale tests and beneficial for senior citizens in the modern era [31]. This study is based on resting-state fMRI data, which is more friendly to the older compared to the task state. Admittedly, it is not easy to obtain fMRI measurements for low-income and underdeveloped area groups at this stage. However, considering the objectivity of fMRI in judging the cognitive level of healthy older adults and the non-diagnostic nature of this study, based on this, we developed a model to explore the functional differences in distinguishing the brain regions with high and low cognitive levels in healthy older adults, and to provide our own research ideas for the fMRI study. In addition, with the advancement of technology and the full popularity of MRI medical treatment in the future, it is believed that non-invasive diagnosis based on fMRI will be extremely convenient.

**Table 3:** Comparison of other relevant methods with this study

Article	Methods	CA
Benesty [28]	FC	71.43%
Goryawala [29]	MRI	73.6%
Zhu [30]	SVM	81.33%
Ranran [32]	MIC + SVM	80.1%
Ding [33]	EEG	80.08%
Jiang [34]	EEG	84.5%
**Our model**	**rs-fMRI**	**86.7%**

### Limitations

There were some limitations in the data of healthy young people selected for modeling in the approach presented in this paper. Although, the older adults with different cognitive scores were from the same database, it is ideal that the rs-FCSM required all data from same database, including same race populations. Despite we tried hard to choose the ComBat algorithm for multi-center FC homogenization and near homologous race between the aging dataset and the youth dataset, the exact computing of the rs-FCSM could be affected, leading to be an additional limitation to this study. Furthermore, older public database used in this study only discloses fMRI data under the AAL template, but this template is relatively rough in functional segmentation. In the future, more comprehensive data of linking cognitive performance and aging brain will be coming up to study the sensitivity of the rs-FCSM to cognitive scores of the aging on finely divided functional templates, such as Yeo 400 atlas, Power 256 atlas, etc.

## Conclusions

The rs-FCSM could extract scientific functional biomarkers sensitive to cognitive scores among healthy older individuals in the frontal, temporal, and parietal cortices. These biomarkers could distinguish excellent cognitive ability from poor cognitive ability with 86.67% accuracy of machine-aided classification. Thereby, our work might provide objective metrics for replacing the conventional scale tests.

## Data Availability

The datasets used and/or analysed during the current study are available from the corresponding author on reasonable request.

## Abbreviations

FC: Functional connectivity
rs-FCSM: Resting-state functional connectivity scores model
AD: Alzheimer’s disease
MRI: Magnetic resonance imaging
fMRI: Functional magnetic resonance imaging
rs-fMRI: Resting-state functional magnetic resonance imaging
GSP: Brain Genomics Superstructure Project
WM: White matter
AAL: Automatic Anatomical Labeling
CDI: Connectome distinctiveness index
ROI: Regions of interest
ROC: Receiver operating characteristic
ELM: Extreme learning machine
CA: Classification accuracy
EMCI: Early mild cognitive impairment
LMCI: Late mild cognitive impairment
MIC: Maximal information coefficient
SVM: Support vector machine
